# The Metabolomic Profile of the Essential Oil from *Zanthoxylum caribaeum* (syn. *chiloperone*) Growing in Guadeloupe FWI using GC × GC-TOFMS

**DOI:** 10.3390/metabo12121293

**Published:** 2022-12-19

**Authors:** Lea Farouil, Ryan P. Dias, Gianni Popotte-Julisson, Garrick Bibian, Ahissan Innocent Adou, A. Paulina de la Mata, Muriel Sylvestre, James J. Harynuk, Gerardo Cebrián-Torrejón

**Affiliations:** 1 Université des Antilles, Laboratoire COVACHIM-M2E EA 3592 Campus de Fouillole, UFR SEN, BP 250, CEDEX, 97157 Pointe-à-Pitre, France; 2 Department of Chemistry, University of Alberta, Edmonton, AB T6G 2N4, Canada

**Keywords:** antimetabolites, essential oil, GC × GC-TOFMS, metabolomics, Rutaceae, *Zanthoxylum caribaeum* (syn. *chiloperone*)

## Abstract

The essential oil (EO) from the leaves of *Zanthoxylum caribaeum* (syn. *Chiloperone*) (Rutaceae) was studied previously for its acaricidal, antimicrobial, antioxidant, and insecticidal properties. In prior studies, the most abundant compound class found in leaf oils from Brazil, Costa Rica, and Paraguay was terpenoids. Herein, essential oil from the leaves of *Zanthoxylum caribaeum* (prickly yellow, bois chandelle blanc (FWI), peñas Blancas (Costa Rica), and tembetary hu (Paraguay)) growing in Guadeloupe was analyzed with comprehensive two-dimensional gas chromatography coupled to time-of-flight mass spectrometry (GC × GC-TOFMS), and thirty molecules were identified. A comparison with previously published leaf EO compositions of the same species growing in Brazil, Costa Rica, and Paraguay revealed a number of molecules in common such as β-myrcene, limonene, β-caryophyllene, α-humulene, and spathulenol. Some molecules identified in *Zanthoxylum caribaeum* from Guadeloupe showed some antimetabolic effects on enzymes; the in-depth study of this plant and its essential oil with regard to metabolic diseases merits further exploration.

## 1. Introduction

Medicinal plants contribute significantly to primary health care due to cultural traditions [1] and the lack of access to and affordability of pharmaceuticals. In addition, natural products are potential sources of new and selective agents for the treatment of important tropical diseases [2,3,4]. Nowadays, the search for effective molecules for chronic disease treatments, such as metabolic disorders, has increased, and plants may offer innovative approaches to treat and prevent diseases [5].

*Zanthoxylum* is a genus of Rutaceae, a widely studied family having a worldwide distribution mainly in tropical and subtropical regions. Many species of the *Zanthoxylum* genus have been extensively studied due to their large role in the traditional medicine of various cultures [6,7,8]. These species have demonstrated larvicidal, antiinflammatory, analgesic, antinociceptive, antioxidant, antibiotic, hepatoprotective, antiplasmodial, cytotoxic, antiproliferative, anthelminthic, antiviral, anticonvulsant, and antifungal properties [9]. The extracts and essential oils (EOs) derived from these plants have been used to test for these properties. For example, canthin-6-ones in *Zanthoxylum chileperone* (prickly yellow, bois chandelle blanc (FWI), peñas Blancas (Costa Rica), and tembetary hu (Paraguay)) grown in Paraguay was assessed for the treatment of mice with Chagas disease. An extract of *Zanthoxylum chileperone* stem bark demonstrated anti*Trypanosomacruzi* activity [7]. The reported variety of beneficial properties in the *Zanthoxylum* species has motivated past studies of *Zanthoxylum caribaeum* in Brazil, Colombia, Costa Rica, and Paraguay [10,11,12,13,14,15,16,17,18]. Across these studies, *Zanthoxylum caribaeum* exhibited acaricidal, antimicrobial, antioxidant, and insecticidal activities. To the authors’ best knowledge, there has not been an investigation into the bioactivity or metabolome of *Zanthoxylum caribaeum* (yellow prickly) grown in Guadeloupe.

Some of the aforementioned beneficial properties in *Zanthoxylum* species are due to volatile organic compounds (VOCs). VOC extraction from various sample types was achieved with headspace (HS) sampling [19,20,21]. VOCs could be readily extracted with full automation, no solvent, and a relatively small amount of sample. Often, headspace solid-phase microextraction (HS-SPME) has been selected for volatilomics studies [22,23,24]. HS-SPME is coupled to gas chromatography (GC) for the separation of VOCs in a mixture and the subsequent detection. In some separations, VOCs with similar partition coefficients co-elute, leading to peak assignment errors and a hindrance in compound identification due to poor mass spectral quality. To overcome co-elutions and increase sensitivity in GC, comprehensive two-dimensional gas chromatography (GC × GC) was developed to enhance separation efficiency for complex mixtures [25,26]. HS-SPME coupled to GC × GC yields an increased number of identifiable compounds in a VOC mixture including EOs.

Here, we report the metabolomic profile of the EO of yellow prickly ash, or *Zanthoxylum caribaeum* Lam. (ZC), grown in Guadeloupe. This plant is of interest for its composition of alkaloids, flavonoids, tannins, terpenoids, and so on [7,14,16,27,28,29,30,31,32]. These compounds confer several pharmacological properties [11,14,16,28,30,31,32,33]. We focused on the EO composition of ZC growing in Guadeloupe (FWI), which has not been studied before, and compared it with the EO composition of the same species growing in Brazil, Costa Rica, and Paraguay [10,11,12,13,14,15,16]. In this work, GC × GC-TOFMS was applied to maximize the number of compounds recovered in the EO compared to the previous studies. Similar to the prior studies in Brazil, Costa Rica, and Paraguay, a variety of chemical classes were detected and identified in the metabolomic profile. A number of the identified compounds from prior studies were detected in the current study, while others were notpreviously reported in the literature. This work also focused on the biological effects of select, specific molecules identified in ZC grown in Guadeloupe that align with current knowledge. Some of the reported compounds may exhibit antimetabolic properties, which merits the further study of ZC from Guadeloupe.

## 2. Materials and Methods

### 2.1. Plant Material

**Figure 1:**
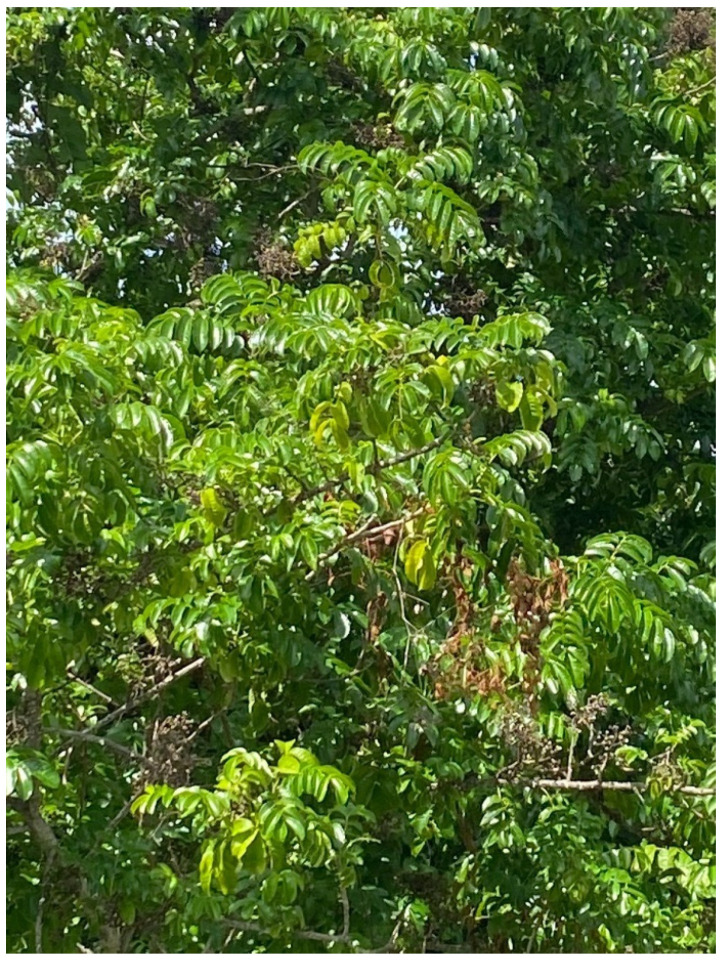
Picture of *Zanthoxylum caribaeum* (Author: L. Farouil).

*Zanthoxylum caribaeum* Lam. leaves (voucher COVA-AI-2022) were collected from a non-reproductive individual tree growing in Gosier Bas-du-fort (16.21725° N, 61.52029° W), Guadeloupe in January 2022 (Figure 1).

### 2.2. Essential Oil

The fresh leaves (751 g) were washed with distilled water, chopped, and hydrodistilled for 2 h 20 min using a Clevenger hydrodistillation apparatus coupled with a microwave reactor (ETHOS X, Milestone^®^, Sorisole, Italy). This setup allowed for heating at 100 °C with a power of 1700 watts. The essential oil was collected and then stored under refrigeration and in the dark at an average temperature of 4 °C until it was analyzed.

### 2.3. HS-SPME-GC × GC-TOFMS

The ZC essential oil (20 µL) was added to 20 mL headspace vials (VWR, CA), and the vials were capped with magnetic screw caps containing septa (Canadian Life Sciences, CA, Canada). Headspace solid-phase microextraction (HS-SPME) was accomplished using an automated SPME module (Gerstel, Linthicum, MD, USA). A tri-mode fibre (50/30 µm DVB/CAR/PDMS; divinylbenzene/carboxen on polydimethylsiloxane, Millipore Sigma, St. Louis, MO, USA) was used to extract the headspace above the EO samples. The fibre was initially conditioned according to the manufacturer’s guidelines. The EO samples were incubated at 60 °C for 5 min to allow for the volatile organic compounds (VOCs) to partition into the headspace. The headspace was extracted for 20 min while the sample was heated at 60 °C. Fibre desorption was achieved by maintaining 250 °C at the septumless head (SLH) of the Cooled Injection System-Programmable Temperature Vaporizing (CIS-PTV) inlet (Gerstel) for 180 s. The GC × GC-TOFMS system consisted of an Agilent 7890 (Agilent Technologies, Palo Alto, CA, USA) gas chromatograph and a Pegasus 4D TOFMS (LECO, St. Joseph, MI, USA) with a quad jet liquid nitrogen-cooled thermal modulator. The first dimension (1D) column was a 5% phenyl polysilphenylene-siloxane phase (Rtx^®^-5MS; 60 m × 0.25 mm i.d.; 0.25 µm film thickness) connected with a SilTiteTM µ-Union (Trajan Scientific and Medical, Victoria, Australia) to a second dimension (2D) trifluoropropylmethylpolysiloxane-type phase (Rtx-200; 1.6 m × 0.25 mm i.d.; 0.25 µm film thickness). All columns were from the Restek Corporation (Restek Corp., Bellefonte, PA, USA). The 2D column was installed in a separate oven located inside the main GC oven. The carrier gas was helium at a corrected constant flow rate of 2 mL/min, and the injector operated in the splitless mode. The main oven temperature program was 40 °C (3 min hold) with a ramp of 3.5 °C/min to 190 °C (no hold) and a final ramp of 15 °C/min to 290 °C (12 min hold). The secondary oven was programmed with a constant +5 °C offset relative to the primary oven. The modulation period was 2.50 s (0.40 s hot pulse and 0.85 s cold pulse) with a +15 °C offset relative to the secondary oven. Mass spectra were acquired in the range of m/z 40–800 for 200 spectra/s. The ion source temperature was set at 200 °C, and the transfer line temperature was set at 240 °C. The detector voltage was run at an offset of −200 V relative to the tuning potential, and the ionization electron energy (EI source) was set at 70 eV. Samples were acquired using LECO ChromaTOF^®^ software version 4.72.0.0(LECO, St. Joseph, MI, USA). The data were processed using LECO ChromaTOF^®^ software version 4.71.0.0(LECO, St. Joseph, MI, USA). The expected peak width settings in the 1D and 2D were 12.5 s and 0.16 s, respectively. The peaks were detected from the raw chromatogram using a minimum signal-to-noise (S/N) ratio value of 100 with a minimum sub-peak S/N of 6. The minimum match required to combine sub-peaks was 600. The identities of the peaks were tentatively assigned based on mass spectral similarity matches (>700) against library spectra and temperature-programmed retention index matches (±15 RI window; when available in PubChem). The retention indices (RIs) were calculated using *n*-alkanes from C7-C22 in a separate analysis under the same extraction and GC × GC-TOFMS conditions. Mass-spectral library searches were performed against the NIST/EPA/NIH Mass Spectral Library (NIST 17) and Wiley Registry of Mass Spectral Data (9th edition). The peak areas were integrated with unique *m*/*z*. The filtering scripts for the GC × GC-TOFMS data [34] automatically and rapidly identified the chemical classes of interest.

## 3. Results and Discussion

### 3.1. HS-SPME-GC × GC-TOFMS Analysis and Comparison

**Figure 2:**
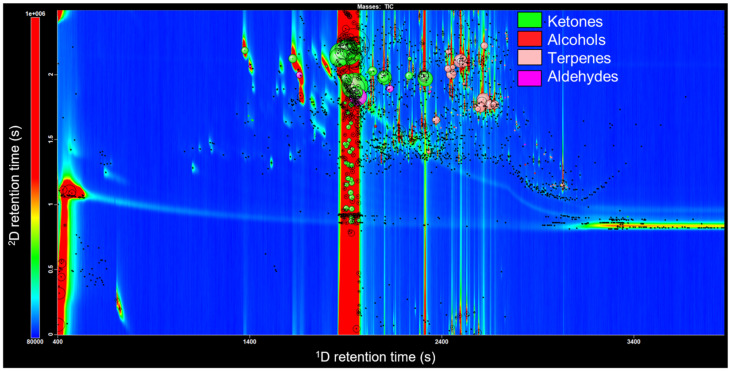
GC × GC-TOFMS total ion chromatogram (TIC) contour plot obtained from *Z. caribaeum* essential oil with filtering scripts applied [34].

The goal of this study was to compare the volatile metabolite profile of the essential oil of yellow prickly ash (*Z. caribaeum*) grown in Guadeloupe (Figure 1) to the ZC essential oils from other regions. Herein, GC × GC-TOFMS was used to facilitate the detection of the previously identified *Z. caribaeum* VOCs and the uncharacterized VOCs of potential interest. Automated filtering scripts for the GC × GC-TOFMS data were applied to the obtained chromatograms to classify the chemical families. GC × GC-TOFMS chromatograms could be read similarly to a contour map. The colors emerge at the reader based on the color scale on the left of the image. Dark blue denotes the baseline or background, while red denotes high intensity (i.e., amount). Each peak or “blob” that the reader sees represents one compound on the two-dimensional plot. A chromatogram with script bubbles is provided in Figure 2.

**Figure 3:**
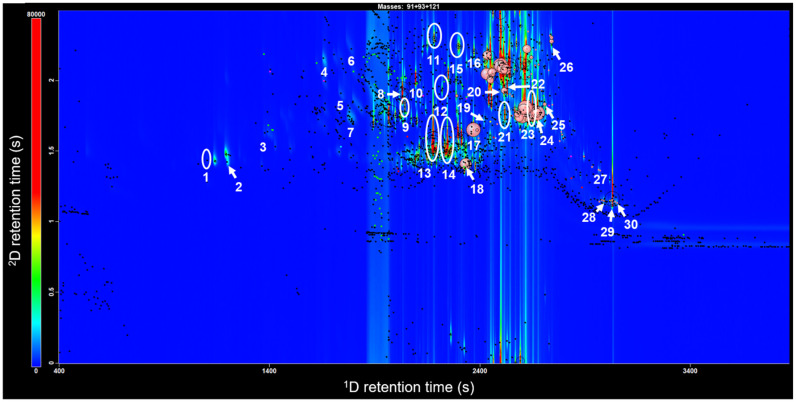
GC × GC-TOFMS extracted ion chromatogram (EIC) contour plot obtained from *Z. caribaeum* essential oil with the tentatively identified compounds labelled. The extracted masses are terpenoid ions *m*/*z* = 91, 93, and 121.

Filtering scripts are able to classify four major chemical families: alcohols, aldehydes, ketones, and terpenoids. The large band in the middle of the chromatogram is 2-undecanone, a ketone in such high abundance that detection yields a large streak. Other ketones, alcohols, aldehydes, and terpenoids were present in the essential oil but in far lesser amounts. Terpenoids were the most common compound class detected with the GC × GC-TOFMS, and were subject to further exploration. A number of terpenoids were tentatively identified based on the MS and RI matches, and a labelled extracted ion chromatogram is shown in Figure 3.

The essential oil of the fresh leaves of ZC was recovered in a 0.06% yield, and the terpenoid compounds are displayed in Table 1. Thirty terpenoids were tentatively identified and numbered according to their retention orders in Figure 3 and Table 1. The relative amounts of each terpenoid are provided as a percentage of the total identified terpenoid peak areas.

The three most abundant compounds of the thirty identified terpenoids were β-caryophyllene (33.23%), cis-carvyl acetate (18.88%), and β-damascenone (10.78%). Other identified terpenoids ranged from 0.03% to 5.20% of the total terpenoid peak area. One-third of the identified terpenoids are common with ZC’s leaves’ EO from different tropical countries (Paraguay [10], Costa Rica [15], and Brazil [11,12,13,14,16]). Limonene and β-caryophyllene were present in ZC’s leaves’ EO in all regions. Limonene was reported with a low yield of 0.3% and 0.42% in the EO species located in Paraguay [10] and Brazil [12], whereas this molecule was one of the most abundant compounds identified in the species from Costa Rica with a yield of 16.7% [15]. Unlike the current work, β-caryophyllene was reported with a low yield of 0.5% by Guy et al. [10] (Paraguay) and an approximate 4% yield in species located in Costa Rica [15] and Brazil [11,13,14]. β-myrcene, α-humulene, α-selinene, and spathulenol were also reported many times in the EOs from these three different countries. Caryophyllene oxide molecules occurred at least once in ZC’s leaves’ EO located in Paraguay. In the current work, nearly twenty molecules are either not listed elsewhere or present in another form, i.e., a different oxidation state or a different isomer. α-muurolene was identified as Γ-muurolene in the species located in Brazil [11,13,14], and 3-eudesmen-11-ol was identified as eudesm-11-en-1-ol by Souza et al. [16]. This comparison shows that the same species has a very high variability in its composition across origins.

### 3.2. Antimetabolic Effects of Some Molecules

**Figure 4:**
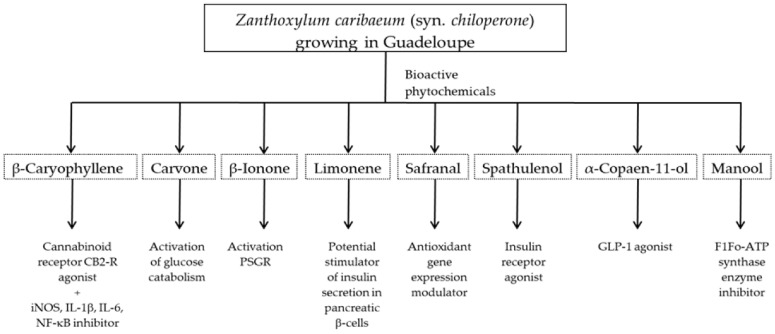
Antimetabolic mechanisms of the bioactive phytochemicals from *Zanthoxylumcaribaeum* growing in Guadeloupe.

Several studies have shown the biological properties of ZC extracts [35], but very few were interested in the antimetabolic properties of the essential oils extracted from the leaves. Some molecules identified in this article were studied for their interesting properties (Table 2) and mechanistic action on organisms (Figure 4).

A recent review conducted by Francomano et al. showed the great biological potential of β-caryophyllene [36]. It is a selective phytocannabinoid agonist of the type 2 receptor CB2, one of the two classical cannabinoid receptors. Among its various biological activities, β-caryophyllene exerts an antiinflammatory action via inhibiting the main inflammatory mediators, such as inducible nitric oxide synthase, interleukin 1 beta, interleukin-6, tumor necrosis factor-alfa, and so on [37]. Several in vitro and in vivo studies have suggested that treatments with β-caryophyllene improved the phenotype of animals used to model various inflammatory pathologies, such as nervous system diseases (Parkinson’s disease, Alzheimer’s disease, multiple sclerosis, amyotrophic lateral sclerosis, and stroke), atherosclerosis, and tumors (colon, breast, pancreas, lymphoma, melanoma, and glioma cancer). Further insights and clinical trials are required for future human applications. To our knowledge, there are no biological studies investigating cis-carvyl acetate and β-damascenone, but compounds with similar structures, carvone and β-ionone respectively, have interesting biological properties. Bouyahya et al. reviewed the health benefits and pharmacological properties of carvone [38]. This molecule has also demonstrated an antidiabetic effect through its role in the prevention of obesity and metabolic problems associated with high-fat diets. This effect is achieved by improving glycoprotein component abnormalities and controlling glucose metabolism. The potential of β-ionone and related compounds as anticancer agents was reviewed by Ansari et al. [39]. Antitumor activities of β-ionone were demonstrated in melanoma, breast cancer, and chemical-induced rat carcinogenesis. Prostate-specific G protein-coupled receptor (PSGR) activation with its ligand, β-ionone, could suppress prostate cancer cell growth in both in vitro and in vivo models, according to the study by Xie and co-workers [40]. Due to its ability to selectively kill tumor cells and its anti-metastatic and apoptosis induction properties obtained with in vitro and in vivo studies, this molecule and its related compounds could be considered novel candidates in chemopreventive and chemotherapeutic strategies for overcoming cancerous diseases. Along with these potential applications, antiinflammatory, antibacterial, antifungal, and antileishmanial properties were also reported [41].

Although other molecules are present in lower proportions in our study, they have created great interest due to their effects on metabolism. For example, Murali et al. studied the antidiabetic effect of D-limonene in streptozotocin-induced diabetic rats [42]. This molecule was reported to have a number of pharmacological effects, including antioxidant, chemopreventive, and anticarcinogenic properties. A 100 mg/kg body weight D-limonene dose shows more effect on increasing plasma glucose and glycosylated hemoglobin levels. Glucose 6-phosphatase and fructose 1, 6-bisphosphatase enzyme activities were increased, while glucokinase activity was decreased along with liver glycogen in diabetic rats. These results suggest the potential antihyperglycemic activity of D-limonene. Safranal, only identified in ZC’s leaves’ EO from Guadeloupe, was reported for its antioxidant properties and ability to improve chemically-induced diabetes [43].This compound modulates antioxidant gene expression and upregulates mitochondrial antioxidant genes, leading to a lower mitochondrial oxygen radical generation. This may, in part, induce an improvement in hyperglycemia, hyperlipidemia, and oxidative stress in an experimental model of diabetes [44]. By modulating oxidative stress in streptozotocin-diabetic rats, safranal may also be effective in the treatment of diabetes [45]. Samini et al. reported that safranalmighthave antihyperglycemic effects without hepatic and renal toxicities in alloxan-diabetic rats [44]. Molecular docking studies showed spathulenoland α-copaen-11-ol, compounds identified in *Cinnamomum travancoricum* leaves’ essential oil, play an active role in antidiabetic activity through α-amylase, α-glucosidase, insulin receptors, and insulin secretion protein, GLP-1 [46]. According to Ravera et al., manool could serve as an exogenous antioxidant to prevent oxidative damage in photoreceptors, a risk factor for degenerative retinal diseases. The molecular docking study confirmed that the OxPhos machinery is ectopically expressed in the outer rod segments and that the F1Fo-ATP synthase enzyme is a target of manool, which inhibited the outer segments’ ATP synthesis by binding to the F1 moiety with high affinity [47].

## 4. Conclusions

This is the first time that the yellow prickly ash, or *Zanthoxylum caribaeum* (syn. *chiloperone*), leaves’ essential oil from Guadeloupe is reported in the literature. Its metabolite profile was determined to be similar in composition to the same species located in Brazil, Costa Rica, and Paraguay. Numerous compounds were detected, and thirty terpenoids were tentatively identified using GC × GC-TOFMS. Among them, eight of the identified terpenoids pressented antimetabolic properties in the literature. Two of the identified terpenoids have no reported activity, but their structural analogs exhibit antimetabolic effects. GC × GC-TOFMS facilitated a rapid and simple method for profiling the volatile metabolome of yellow prickly. These results suggest the biological potential of *Zanthoxylum caribaeum*, and provide a preliminary metabolite profile for further study on the essential oil from Guadeloupe. Future work on the metabolomics of the yellow prickly ash should follow this protocol to maximize the number of identifiable VOC metabolites.

## Figures and Tables

**Table 1:** Topological formulae of the molecules present in the *Zanthoxylum caribaeum* essential oil from Guadeloupe.

Number	Name and Topologic Formula	Present in the EO in Other Regions	Retention Index (Observed)	Retention Index (Library)	Forward MS Match	Reverse MS Match	Total Terpenoid % Area
Hydrocarbonatedmonoterpenes							
1	β-Myrcene 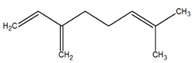	Paraguay [10] Brazil [11,12,13,14]	998	989	743	835	0.33
2	Limonene 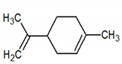	Paraguay [10] Costa Rica [15] Brazil [12]	1034	1034	900	900	0.78
3	p-Cymenene 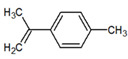		1102	1105	908	914	0.31
Diterpenes							
28, 30	Thunbergene isomers 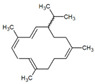		1958, 2041	Not reported	831, 822	833, 824	0.16, 0.14
29	Manool 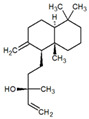		2011	Not reported	774	785	0.80
Hydrocarbonatedsesquiterpenes							
13	β-Caryophyllene 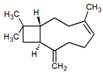	Paraguay [10] Costa Rica [15] Brazil [11,13,14,16]	1445	1446	878	881	33.23
14	α-Humulene 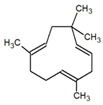	Paraguay [10] Brazil [11,13,14]	1476	1477	976	976	2.54
16	Patchoulane 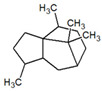		1539	1552	765	768	1.00
18	α-Farnesene 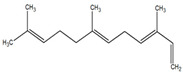		1518	1517	926	930	2.60
20	α-Selinene 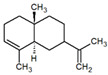	Brazil [11,12,13,14]	1610	Not reported	805	832	0.12
27	α-Muurolene 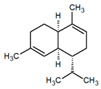		1935	Not reported	703	783	0.04
Oxygenated sesquiterpenes							
17	Spathulenol 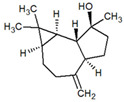	Paraguay [10] Brazil [11,12,13,14,16]	1537	Not reported	799	810	2.42
21	3-Eudesmen-11-ol 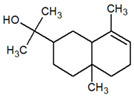		1613	1616	778	778	4.38
23	Caryophyllene oxide 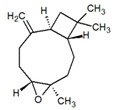	Paraguay [10]	1611	1612	830	830	2.84
24	Isoaromadendrene epoxide 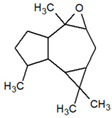		1600	1594	711	753	0.03
25	Ledene oxide (II) 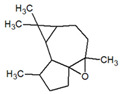		1716	Not reported	804	804	0.53
Other terpenoids							
19	α-Copaen-11-ol 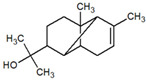		1563	1549	918	918	0.39
26	4,6,6-Trimethyl-2-(3-methylbuta-1,3-dienyl)-3-oxatricyclo[5.1.0.0(2,4)]octane 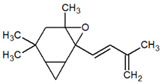		1736	Not reported	807	807	1.01
4	Safranal 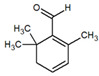		1214	1212	902	905	0.53
5	cis-Verbenone 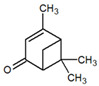		1249	1245	811	816	0.57
6	Carvone 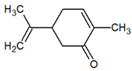		1270	1262	892	892	1.96
10	β-Damascenone 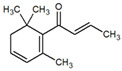		1406	1401	921	921	10.78
11	α-Ionone 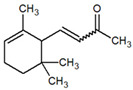		1448	1456	925	925	0.82
12	Geranyl acetone 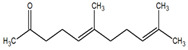		1465	1468	960	964	4.84
15	β-Ionone 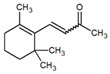		1505	1500	946	946	1.45
22	Germacrone 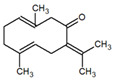		1620	Not reported	825	826	5.20
7	Linalylformate 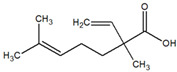		1266	1256	794	804	0.24
8	cis-Carvyl acetate 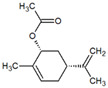		1378	1363	813	813	18.88
9	trans-Carvyl acetate 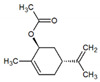		1382	1368	924	924	1.09

**Table 2:** Antimetabolic activities of molecules present in *Zanthoxylum caribaeum* essential oil from Guadeloupe.

Name	Present in the EO in Other Regions	Antimetabolic Properties
β-Caryophyllene	Paraguay [10] Brazil [11,12,13,14]	Antiinflammatory and antitumor
cis-Carvyl acetate		Currently unknown
β-Damascenone		Currently unknown
Carvone		Antidiabetic and obesity prevention
β-Ionone		Anticancer, antitumor, antiinflammatory, antibacterial, antifungal, and antileishmanial
Limonene	Paraguay [10] Costa Rica [15] Brazil [12]	Antidiabetic, antioxidant, and anticarcinogenic
Safranal		Antioxidant, antidiabetic, and antihyperglycemic
Spathulenol	Paraguay [10] Brazil [11,12,13,14,16]	antidiabetic
α-Copaen-11-ol		Antidiabetic
Manool		Antioxidant

## Data Availability

The data presented in this study are available in article.
